# Neurodevelopmental outcomes at five years in children born very preterm (24–31 weeks) exposed to opioids with or without midazolam: results from the French nationwide EPIPAGE-2 cohort study

**DOI:** 10.1016/j.lanepe.2025.101242

**Published:** 2025-02-18

**Authors:** Elizabeth Walter-Nicolet, Laetitia Marchand-Martin, Andrei S. Morgan, Monique Kaminski, Valérie Benhammou, Pierre-Yves Ancel, Pierre Tourneux, Véronique Pierrat, Xavier Durrmeyer

**Affiliations:** a University Paris-Cité, CRESS, Obstétrical Perinatal and Pediatric Epidemiology Research Team, EPOPé, INSERM, INRAE, Paris, France; b Neonatal Intensive Care Unit, Saint Joseph Hospital, 185 rue Raymond Losserand, Paris 75014, France; c Gynecology-Obstetric Department, Max Fourestier Hospital, 403 Avenue de la république, Nanterre 92014, France; d Elizabeth Garrett Anderson Institute for Women’s Health London, University College London, London, UK; e Department of Neonatology, National Maternity Hospital, Holles Street, Dublin, Ireland; f Neonatal and Paediatric Intensive Care Unit, University Hospital, Amiens, France; g PériTox - UMI 01, Medicine University, Picardie Jules Verne University, Amiens, France; h Neonatal Intensive Care Unit, Centre Hospitalier Intercommunal de Créteil, Créteil, France; i Université Paris Est Créteil, Faculté de Santé de Créteil, IMRB, GRC CARMAS, Créteil, France

**Keywords:** Very preterm neonates, Opioids, Midazolam, Cumulative exposure, Five years neurodevelopment, Nationwide cohort study

## Abstract

**Background:**

Data on preschool neurodevelopment of preterm infants according to the duration of their neonatal exposure to opioids with/without midazolam is limited. We aimed to assess neurodevelopment outcome in children aged five years, born very preterm (24–31 weeks), according to exposure to these drugs.

**Methods:**

Secondary analysis from the French prospective cohort study EPIPAGE-2 (Etude Epidémiologique sur les Petits Ages Gestationnels, 2011). Exposure to opioids with/without midazolam was classified as none, ≤7 or >7 days. Percentages were weighted to account for the study design. The primary outcome was moderate/severe neurodevelopmental disabilities (NDD). Analyses were conducted using logistic regression and adjusted for perinatal confounders.

**Findings:**

Among 3117 survivors, 1165 (35.9%) were exposed (762/1165 (68.0%) ≤7 days, 403/1165 (32.0%) >7 days). Of these 49.5% received opioids only, 41.4% opioids and midazolam, and 9.1% midazolam only. Moderate/severe NDD occurred in 17.8%, 18.9%, and 31.7% in the unexposed, exposed ≤7 days, and exposed >7 days groups, respectively. After adjustment for baseline confounders, only exposure >7 days was associated with increased rates of moderate/severe NDD (adjusted odds ratio 2.07; 95% CI 1.32–3.26). After additional adjustment for severe neonatal morbidities no significant association was found between any duration of exposure and NDD.

**Interpretation:**

Exposure to opioids with/without midazolam >7 days might be associated with a higher prevalence of moderate/severe NDD at five years in very preterm born children but severe neonatal morbidities are a major modulator of this association.

**Funding:**

French Institute of Public Health Research, 10.13039/501100001677National Institute of Health and Medical Research, National Institute of Cancer, National Solidarity Fund for Autonomy, PremUp, APICIL Foundations.

**Research in context**

**Evidence before this study**

Despite concerns about their potential neurotoxicity to the immature brain and their effect on outcome, the use of opioids and/or benzodiazepines to manage pain and stress is standard practice in neonatal care. It is therefore of great importance to evaluate exposure to these drugs during the neonatal period and subsequent neurodevelopment. We searched Pubmed for articles published in English up to November 2024 using the terms “opioids”, “morphine”, “fentanyl”, “sufentanil”, “benzodiazepines”, “midazolam”, “preterm neonates”, “neurodevelopmental outcome”, “immature brain”. There is increasing evidence that the cumulative duration and/or dose of these drugs may be associated with adverse neurodevelopmental outcomes in early childhood, up to two years of age. Data on outcomes in the preschool years are controversial, either published before the widespread use of opioids/benzodiazepines or based on retrospective single-centre studies. The aim of this study was to investigate the association between neonatal exposure to opioids with/without midazolam, duration of exposure, and neurodevelopmental outcomes at five years in a large population-based cohort of children born very preterm.

**Added value of this study**

The findings, based on prospectively collected data from more than 3000 children born very preterm (<32 weeks’ gestational age (GA)), indicate that cumulative exposure of more than seven days is associated with higher odds ratios of mild, moderate/severe neurodevelopmental impairment at five years, mainly related to a reduction in total full-scale intelligence quotient. The combination of opioids and midazolam was associated with the worst adverse effects. In exploratory subgroup analyses, the results were more pronounced in male children and children born extremely preterm (24–27 weeks’ GA) were no more vulnerable than those born very preterm (28-21 weeks’ GA). However, following additional adjustment for severe neonatal morbidities, there was no longer any association between exposure lasting more than seven days and subsequent neurodevelopment. This suggests that not only the type or duration of exposure, but also associated morbidities during the neonatal period – that can influence and/or result from the use of the studied drugs – need to be considered when analysing pain management practices and long-term neurodevelopmental outcomes in children born preterm.

**Implications of all the available evidence**

Opioids with/without midazolam should be used with caution in very preterm neonates, as prolonged exposure may be associated with an increased risk of adverse neurodevelopmental outcomes in the preschool years, mainly in the cognitive domain. A global strategy, including non-pharmacological approaches and family-centred care, should be considered for every preterm neonate to reduce both exposure to painful procedures and the use of such treatments. In cases where the patient's condition requires prolonged use of these drugs, it is essential to monitor the child's neurodevelopment closely and to inform the parents. The efficacy and safety of new drugs that could provide analgesia and/or sedation should be investigated.

## Introduction

Although survival of extremely (<28 weeks' gestational age (GA)) and very preterm (28–32 weeks' GA) neonates has improved in recent decades, these children remain at high risk of later neurodevelopmental delay.^1^^,^^2^ The causes of these impairments are interrelated, making it difficult to distinguish between neonatal events or treatments and later neurodevelopment. The invasive, painful procedures that neonates undergo are critical for their survival,^3^ but they can also affect their future development.^4^^,^^5^ Morphine and synthetic opioids can be used to alleviate pain and stress during invasive procedures or surgery, while benzodiazepines can be used to sedate preterm neonates undergoing mechanical ventilation.^6^ However, the benefit-risk balance of these interventions remains uncertain,^6^ leading to large inter-unit variability in opioids and/or benzodiazepines administration rates.^7^, ^8^, ^9^, ^10^ In animal models, morphine alters the growth and survival of Purkinje cells in the developing cerebellum, while midazolam impairs cell growth and maturation in hippocampal, cortical and subcortical regions, both leading to poorer behavioural and cognitive performance.^4^ In humans, studies evaluating the association between neonatal exposure to opioids and/or benzodiazepines and later neurodevelopment are scarce, with conflicting results.^6^, ^7^, ^8^^,^^11^, ^12^, ^13^, ^14^, ^15^, ^16^ In neonates born preterm, morphine and midazolam exposure have been associated with cerebellar and hippocampal dysmaturation, respectively, and altered neurodevelopmental outcomes at later ages.^11^^,^^12^ A recent study using the England and Wales National Neonatal Research Database showed an association between opioid use for more than two consecutive days in ventilated very preterm neonates and an increased risk of brain injury in the first four weeks of life.^13^ At 18–24 months, several studies,^7^^,^^8^^,^^14^ most notably that of Puia-Dimitrescu et al.,^8^ showed that exposure less than or equal to seven days to opioids and/or benzodiazepines in extremely preterm neonates was not associated with worse motor, cognitive or language Bayley scores.^7^^,^^8^^,^^14^ Conversely, exposure longer than seven days was significantly associated with lower scores in all three domains, especially for the opioid–benzodiazepine combination.^8^ At five years, results are controversial.^14^, ^15^, ^16^ The Etude Epidémiologique sur les Petits Ages Gestationnels (EPIPAGE, 1997) showed that exposure more than seven days to opioids and/or sedatives was not associated with poorer cognitive outcome in very preterm neonates.^15^ Two single-centre studies of about 100 very preterm neonates born in the 2000s reported conflicting results.^14^^,^^16^ One study reported a lower Full Scale Intelligence Quotient (FSIQ) associated with a higher cumulative morphine dose,^14^ whereas the other identified an increased risk of autism spectrum features but no cognitive impairment.^16^

To take this a step further in a contemporary large population-based cohort of very preterm neonates, a secondary analysis of the EPIPAGE-2 study was conducted to assess neurodevelopment at five years according to whether or not children were exposed to opioids with/without midazolam during the neonatal period and the duration of such exposure.

## Methods

### Data source and study population

EPIPAGE-2 is a French prospective national population-based cohort of children born preterm.^17^ All births from 22 weeks to 0 day (22 + 0) to 34 weeks-6 days (34 + 6) in all maternity units (n = 546) in 25 French regions were eligible for inclusion. The study lasted from March to December 2011. Neonates born at 22–26 weeks were recruited during an 8-month period (35 weeks) and those born at 27–31 weeks during a 6-month period (26 weeks).^17^ At five years, survivors underwent a planned standardised assessment of their overall neurodevelopment covering four domains: motor, cognitive, behavioural and sensory (Clinicaltrial.gov identifier: NCT03078439).^1^^,^^17^ Motor evaluation screened for cerebral palsy (classified using the Gross Motor Function Classification System (GMFCS)) and for developmental coordination disorders (assessed by the Movement Assessment Battery for Children (M-ABC)).^1^ Cognition was assessed with the French version of the Wechsler Preschool and Primary Scale of Intelligence, fourth edition (WPPSI-4), that gives a composite Full Scale Intelligence Quotient (FSIQ).^1^ Behaviour was assessed through the Strengths and Difficulties Questionnaire (SDQ).^1^ The sensory domain comprised vision and hearing and was assessed through parental reports and clinical examination.^1^ If the assessment team was not available, parents were asked to complete a postal questionnaire including information on cerebral palsy and severe/moderate sensory disabilities.^1^ A composite measure with four levels of neurodevelopmental disabilities (NDD) (none, mild, moderate, severe) was created using the results of these evaluations.^1^

For this secondary analysis, neonates born at 24–31 completed weeks were eligible. We excluded patients with severe congenital cerebral malformation at birth, those with missing data on exposure status, age at treatment initiation or duration of exposure, and those exposed to morphine boluses only, as continuous infusion of analgesia and/or sedation is the preferred modality in France.^10^^,^^18^ We selected maternal, neonatal and unit data collected at birth, and neurodevelopmental data collected at five years.^1^^,^^17^ For mothers, causes of preterm birth were defined as: (1) birth after preterm labour (PL), defined as spontaneous onset of labour before rupture of membranes; (2) birth after preterm rupture of membranes (PROM), defined as spontaneous rupture of membranes occurring at least 12 h before birth. PPROM and PL were distinguished and associated as appropriate with maternal inflammatory response syndrome (MIRS), defined as maternal C-reactive protein (CRP) level 50 mg/L before delivery; (3) vascular placental disease without antenatal diagnosis of intrauterine growth restriction (IUGR), defined as maternal hypertension, preeclampsia, eclampsia, and HELLP syndrome (hemolysis, elevated liver enzymes, and low platelet count); (4) vascular placental disease with an antenatal diagnosis of IUGR, defined as impaired fetal growth with or without abnormal Doppler US findings; (5) isolated antenatal diagnosis of IUGR; (6) isolated and acute placental abruption. Antenatal steroids treatment was considered when the mother received at least one injection of betamethasone. Magnesium sulphate (MgSO4) administration was considered when the mother received at least a loading dose of four grams of MgSO4 before delivery. Socioeconomic status was defined as the highest occupational status of the mother and father, or mother only if a single parent. Neonatal GA (in weeks) at birth, was determined as the best obstetrical estimate combining last menstrual period and first-trimester ultrasonography assessment. Unit characteristics were level of the maternity (level 3 was defined so if maternity wards were associated with a neonatal intensive care unit (NICU)) and neonatal unit volume activity.

We also selected information about five severe neonatal morbidities which are associated with both exposure to opioids and/or midazolam and with later neurodevelopmental outcome: severe cerebral abnormalities (defined as grade III or IV intraventricular haemorrhage (IVH) and/or cystic periventricular leukomalacia (cPVL)),^1^ stage 2 or 3 necrotizing enterocolitis (NEC) (defined according to Bell’s classification),^1^ stage 3 or higher retinopathy of prematurity (ROP),^1^ severe bronchopulmonary dysplasia (BPD) defined as administration of oxygen for at least 28 days plus need for 30% or more oxygen and/or mechanical ventilator support or continuous positive airway pressure at 36 weeks’ postmenstrual age,^1^^,^^17^ and late neonatal sepsis, defined as positive blood culture occurring after 72 h of life, associated with antibiotic administration for 5 days or more, or death within 5 days following positive blood culture^1^^,^^17^ IVH and cPVL were diagnosed by cranial ultrasonography (cUS) images. The standard practice in France was to perform 1 or 2 cUS during the first week after birth and then weekly for the following two weeks. According to the 2011 international recommendations, an additional cUS was usually conducted at the term-corrected age.

### Exposure

Any continuous exposure to opioids (morphine, fentanyl, sufentanil) with/without midazolam (the only benzodiazepine used as a continuous sedative in French Neonatal Intensive Care Units (NICUs) in 2011) was considered as the exposure. We defined three groups according to exposure: unexposed if no exposure was recorded during the NICU stay, exposed less than seven days (≤7 days) if there were seven days or fewer of cumulative exposure, and exposed more than seven days (>7 days) if the total cumulative number of days exposure was more than seven. Age at first exposure and total number of days of exposure to each drug were recorded, but there was no information on whether drugs were given concurrently or not, or on the sequence of administration if several opioids were given during the NICU stay. If multiple drugs were started on the same day, cumulative exposure was determined by the drug prescribed for the longest duration. When treatments started on different days, the cumulative exposure duration was calculated as the longest exposure duration if the exposure periods overlapped, or as the sum of the individual exposures if the periods were completely different. In case of doubtful classification, an individual check was done. Overall, we created three exposure groups: opioids only, midazolam only, and opioids–midazolam combination. The variability of prescriptions by NICU and by GA groups were also described.

### Primary outcome

The primary outcome was the occurrence of moderate/severe neurodevelopmental disabilities (NDD) in survivors at five years. Severe NDD were defined as severe cerebral palsy (GMCSF levels 4–5), and/or bilateral blindness (visual disability <1/10), or deafness (>70 dB) not corrected, or partially corrected with hearing aids, and/or FSIQ < −3 standard deviations (SD).^1^ Moderate NDD were defined as cerebral palsy (GMCSF 2–3), and/or visual disability <3.2/10 but ≥1/10, and/or unilateral or bilateral hearing between 40 and 70 dB, and/or FSIQ between - 3 and −2 SD.^1^

### Secondary outcomes

- Mild NDD, defined as mild cerebral palsy (GMFCS 1), and/or visual disability ≥3.2/10 and < 5/10, and/or hearing loss <40 dB, and/or FSIQ between −2 SD and −1 SD, and/or developmental coordination disorders, defined as an MABC-2 score less than or equal to the fifth centile (≤5th p), and/or behavioural difficulties defined as a total SDQ score greater than or equal to the 90th centile (≥90th p),^1^
- Cerebral palsy (CP),^1^
- FSIQ, MABC-2 and SDQ scores,^1^
- Severe and moderate visual and hearing disability.^1^

### Statistical analysis

Descriptive statistics and graphic illustrations were used to describe the demographic and baseline maternal and neonatal characteristics. Primary analyses were conducted after multiple imputation by chained equations to handle missing data. Variables in the imputation model included both those potentially predicting non-response and those predicting outcomes. Considering missing data as missing at random based on the variables included in the imputation model, we generated fifty independent imputed datasets with thirty iterations each (R package mice).^19^ Estimates were pooled according to Rubin’s rules.^20^ Data and percentages were weighted to take into account the study design which included different inclusion periods according to gestational age (24–26 weeks recruited during 35 weeks, weight 1 (35/35)); 27–31 weeks recruited during 26 weeks, weight 1.35 (35/26).^1^^,^^17^ A regression analysis with cluster-robust standard errors was used to account for the dependence of observations within the same neonatal intensive care units.^21^ All tests were two-sided; a p-value less than .05 was considered statistically significant. No adjustments were made to account for multiple testing. The statistical significance of secondary outcomes and subgroup analyses should therefore be interpreted as exploratory. Statistical analyses were performed using R 4.1.1 software.

### Main analysis

The adjustment factors were selected based on their clinical relevance. Two main models were constructed. The first model (model 1) was adjusted for GA and confounding factors occurring at birth and before the exposure (maternal age at birth, mother born in France, parents’ socio-economic status, cause of preterm birth, antenatal corticosteroids, MgSO4 administration, multiple pregnancy (twins or more), sex, small for GA,^22^ 5-min Apgar score below seven, birth in level 3 unit (maternity beside a neonatal intensive care unit), neonatal unit volume activity). To account for the possibility that the sickest neonates may also be the most exposed, a second model (model 2) was constructed, adjusted for the variables of model 1 and the five severe neonatal morbidities described above.

### Subgroup analyses

To gain further insight into the potential associations between exposure to opioids only, opioids with midazolam exposure, and neurodevelopmental outcomes, we conducted three subgroup analyses using the previously described models and taking into account the duration of exposure. The results are thus presented for children exposed to opioids only, or to opioids with midazolam, then by sex, and finally by GA category (24–27 weeks and 28–31 weeks).

### Sensitivity analysis

As a sensitivity analysis, and to assess the consistency of the results observed with multiple imputation, results on complete cases are reported.

### Ethics approval

As required by French law and regulations, recruitment and follow-ups were approved by the national data protection authority (Commission Nationale de l’Informatique et des Libertés, CNIL DR-2011–089, DR-2012–246, DR-2013–406, DR-2016–290) and by the appropriate ethics committees, i.e., the advisory committee on the treatment of personal health data for research purposes (Comité Consultatif sur le Traitement de l’Information en matière de Recherche, CCTIRS, reference nos. 10–626, 12–109 and 16–263) and the committee for the protection of people participating in biomedical research (Comité de Protection des Personnes, CPP, reference nos. 2011-A00159-32 and 2016-A0033-48).

Recruitment and data collection occurred after the parents received oral information about the study and orally agreed to participate.

### Role of the funding source

The funding source had no role in the design and conduct of the study; collection, management, analysis, and interpretation of the data; preparation, review, or approval of the manuscript; and the decision to submit the manuscript for publication.

## Results

### Cohort characteristics

**Fig. 1:**
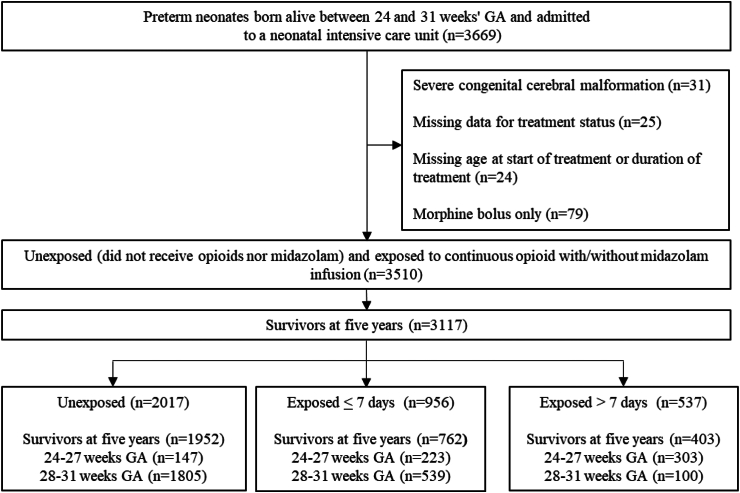
**Population flow chart.** Population percentages were weighted according to varying recruitment periods for those born at 24–26 weeks (35 weeks) and at 27–31 weeks (26 weeks) gestational age. Abbreviations: Gestational age: GA.

Among the 3669 eligible neonates born preterm at 24–31 weeks, 3510 (95.7%) were eligible for the study and 3117, survivors at five years, were included (Fig. 1). From these 3117 survivors, 1883 (60.4%) achieved a complete assessment, 337 (10.8%) a partial assessment (parental questionnaire only), and 897 (28.8%) were lost to follow-up.

Maternal and neonatal characteristics of the 3117 survivors at five years are summarised in Table 1. Neonates exposed for more than seven days were more likely to have a 5-min Apgar score below seven, to be born extremely preterm, and to have at least one severe neonatal morbidity (Table 1). Characteristics of participating and non-participating children at five years are presented in Supplementary Table S1. Non-participating children, or children with incomplete assessments, were more often from disadvantaged families, but there were no differences in neonatal characteristics, treatment allocation or severe neonatal morbidities between the two groups (Supplementary Table S1).

**Table 1:** Maternal, neonatal and units’ characteristics according to exposure and duration of exposure to opioids with/without midazolam, among survivors at five years.

	Unexposed (n = 1952)	Exposed		p value
		≤7 days (n = 762)	>7 days (n = 403)	
Maternal characteristics at birth				
Maternal age				
<25 years	368/1952 (18.8)	147/762 (19.5)	70/403 (17.3)	0.61
25–34 years	1157/1952 (59.3)	460/762 (60.1)	235/403 (58.3)	
≥35 years	427/1952 (21.9)	155/762 (20.4)	98/403 (24.4)	
Birth in France	1496/1927 (77.7)	571/754 (75.6)	283/401 (70.6)	0.011
Parents’ socio-economic status^a^				
Executive	364/1843 (19.9)	181/729 (24.7)	87/376 (23.0)	0.33
Intermediate	390/1843 (21.2)	145/729 (20.0)	69/376 (18.3)	
Administration	515/1843 (28.0)	189/729 (26.1)	103/376 (28.2)	
Service, trade	271/1843 (14.5)	101/729 (13.8)	56/376 (14.7)	
Worker, unemployed	303/1843 (16.4)	113/729 (15.4)	61/376 (15.7)	
Multiple pregnancy	628/1952 (32.3)	249/762 (32.6)	135/403 (33.0)	0.97
Cause of preterm birth				
Preterm labour	747/1952 (38.1)	311/762 (40.1)	159/403 (37.6)	0.41
Preterm premature rupture of membranes	490/1952 (24.8)	157/762 (20.1)	100/403 (24.6)	
Hypertensive disorders or placenta abruption	441/1952 (22.9)	185/762 (25.0)	89/403 (23.4)	
Isolated foetal growth restriction	126/1952 (6.6)	44/762 (6.0)	22/403 (6.2)	
Other	148/1952 (7.6)	65/762 (8.7)	33/403 (8.2)	
Antenatal corticosteroids	1633/1915 (85.3)	626/748 (83.5)	334/399 (84.3)	0.49
Magnesium sulphate administration	153/1923 (8.0)	70/749 (9.4)	44/397 (11.6)	0.061
Neonatal characteristics				
Gestational age (weeks)				
24	8/1952 (0.3)	10/762 (1)	34/403 (7.4)	<0.001
25	41/1952 (1.6)	47/762 (4.8)	86/403 (18.6)	
26	98/1952 (3.8)	74/762 (7.5)	113/403 (24.5)	
27	148/1952 (7.7)	92/762 (12.6)	70/403 (20.4)	
28	233/1952 (12.2)	120/762 (16.5)	39/403 (11.4)	
29	311/1952 (16.2)	133/762 (18.3)	25/403 (7.3)	
30	466/1952 (24.3)	143/762 (19.6)	18/403 (5.2)	
31	647/1952 (33.8)	143/762 (19.6)	18/403 (5.2)	
Sex, male	986/1952 (50.6)	399/762 (52.5)	213/403 (53.0)	0.52
Small-for-GA^b^	701/1952 (36.3)	244/762 (32.8)	150/403 (39.5)	0.063
Apgar at 5 min < 7	221/1873 (11.7)	159/728 (21.7)	106/357 (29.1)	<0.001
Neonatal morbidities, at least one	399/1868 (20.7)	255/723 (33.7)	292/386 (74.9)	<0.001
Severe cerebral abnormalities	62/1924 (3.2)	52/753 (6.7)	49/402 (12.1)	<0.001
Necrotizing enterocolitis	35/1932 (1.8)	31/752 (4.0)	43/402 (11.4)	<0.001
median [IQR] age necrotizing enterocolitis	21 [12–32]	28 [21–37]	23 [17–45]	
Severe bronchopulmonary dysplasia	61/1914 (3.0)	42/735 (5.3)	135/381 (35.0)	<0.001
Severe retinopathy of prematurity	6/1936 (0.3)	4/756 (0.4)	29/400 (6.5)	<0.001
Late onset sepsis	288/1938 (14.4)	182/750 (23.0)	191/390 (47.6)	<0.001
Median [IQR] age late onset sepsis	12 [8–19]	14 [10–23]	12 [8–23]	
Units’ characteristics				
Birth in level 3^c^	1697/1952 (86.8)	645/762 (84.4)	353/403 (87.9)	0.44
Neonatal unit volume activity^d^				
<55	768/1952 (39.5)	249/762 (33.0)	121/403 (30.2)	<0.001
[55–70]	399/1952 (20.4)	164/762 (21.8)	114/403 (28.4)	
[70–90]	305/1952 (15.8)	144/762 (18.5)	70/403 (17.9)	
≥90	480/1952 (24.3)	205/762 (26.7)	98/403 (23.5)	

Data are No./total (%), unless otherwise indicated. Denominators vary according to the number of missing data for each variable. Percentages are weighted to consider the differences in survey design between gestational age groups, resulting in numbers different from No./total calculation.

GA = gestational age.

a Defined as the highest occupational status between occupations of the mother and the father, or mother only if living alone.

b Small-for-GA was defined as birth weight less than the 10th percentile for GA and sex based on French intrauterine “EPOPé” growth curves.^22^

c Maternity wards were classified as level 3 when associated with a neonatal intensive care unit (NICU).

d Number of neonates born before 32 GA admitted in 2011, obtained from the national hospital discharge database.

The unexposed group exhibited a higher five-year survival rate than the exposed groups (Supplementary Table S2). This was primarily attributable to elevated rates of in-hospital mortality in both exposed groups. The median (interquartile rate, (IQR)) age at death for neonates exposed for more than seven days was higher than that of unexposed neonates (19 (13–32) days and 3 (1–9) days, respectively) (Supplementary Table S2). The causes of death differed between the unexposed and exposed neonates. The unexposed neonates died within the first three days, mainly from respiratory distress syndrome. In contrast, the exposed neonates died from causes that usually occur after the first three days.

Less than 1% of deaths occurred between hospital discharge and five years in all groups (Supplementary Table S2).

### Exposure to drugs of interest

**Fig. 2:**
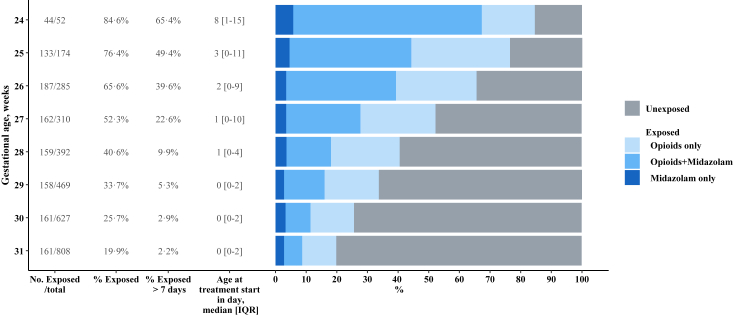
**Drug(s) exposure by gestational age, among survivors at five years**.

In the 3117 children, 1952 (64.1%) were unexposed and 1165 (35.9%) were exposed (Fig. 1). Exposed neonates received opioids only (566 (49.5%)), opioids with midazolam (496 (41.4%)), or midazolam only (103 (9.1%)). Of the 1062 neonates exposed to opioids, 688 (64.7%) received sufentanil, 391 (36.0%) received morphine, and 183 (17.1%) received fentanyl. Various combinations of treatments were observed but among exposed neonates, around one out of two received sufentanil only (Supplementary Table S3). Exposition varied with GA, with 62.7% neonates born extremely preterm exposed, compared to 27.8% of those born very preterm (Fig. 2). The 526 extremely preterm neonates mainly received opioids with midazolam (278/526, 52.2%), then opioids only (216/526, 41.6%) and then midazolam only (32/526, 6.2%). The 639 very preterm neonates received mainly opioids only (350/639, 54.8%), then opioids with midazolam (218/639, 34.1%), and then midazolam only (71/639, 11.1%).

A total of 106 NICUs participated in the study, all of which managed very preterm neonates, while extremely preterm neonates were managed in 66 of them. There was considerable variation in the use of opioids and midazolam between sites (Supplementary Fig. S1). The mean exposure rate across all sites was 39.3%, with a minimum of 0% and a maximum of 82.9%.

### Neurodevelopmental outcomes at five years by exposure duration

#### Primary outcome

Moderate/severe NDD occurred in 17.8%, 18.9% and 31.7% in the unexposed, ≤7 days and >7 days groups, respectively (Table 2). After adjustment for GA and the aforementioned confounders (model 1), there was an association between exposure >7 days and the occurrence of moderate/severe NDD (adjusted odds-ratio (aOR) 2.07; 95% confidence intervals (95% CI) 1.32–3.26) (Table 2). Following additional adjustment for severe neonatal morbidities (model 2), the association between exposure >7 days and moderate/severe NDD at five years was no longer significant (Table 2).

**Table 2:** Neurodevelopmental outcome at five years according to exposure and duration of exposure to opioids with/without midazolam.

	Unexposed (n = 1952), %	Exposed ≤7 days (n = 762), %	Exposed >7 days (n = 403), %	p value	Model 1: aOR or adjusted mean difference (95% CI), vs Unexposed^a^		Model 2: aOR or adjusted mean difference (95% CI), vs Unexposed^a^	
					Exposed ≤7 days	Exposed >7 days	Exposed ≤7 days	Exposed >7 days
Neurodevelopmental disabilities^b^								
None	43.8	40.6	26.2	<0.001	1 [Reference]	1 [Reference]	1 [Reference]	1 [Reference]
Mild	38.4	40.6	42.1		1.07 (0.84–1.37)	1.47 (1.02–2.12)	1.07 (0.84–1.37)	1.37 (0.94–2.01)
Moderate or Severe	17.8	18.9	31.7		1.03 (0.76–1.41)	2.07 (1.32–3.26)	0.98 (0.71–1.34)	1.43 (0.88–2.32)
Cerebral palsy	5.3	6.3	11.4	<0.001	0.99 (0.66–1.49)	1.45 (0.91–2.31)	0.85 (0.54–1.35)	0.85 (0.50–1.45)
Full scale intelligence quotient (FSIQ)								
mean (SD)	94.4 (15.3)	94.0 (15.9)	87.6 (16.6)	<0.001	0.10 (−1.5 to 1.7)	−4.1 (−6.6 to −1.7)	0.32 (−1.3 to 1.9)	−2.3 (−4.9 to 0.16)
< −2 SD^c^	15.0	15.3	27.2	<0.001	0.95 (0.71–1.27)	1.58 (1.05–2.38)	0.90 (0.67–1.21)	1.14 (0.72–1.80)
Developmental Coordination Disorders, MABC-2 score^d^								
mean (SD)	9.5 (3.8)	9.4 (3.8)	8.4 (3.7)	0.007	−0.01 (−0.40 to 0.38)	−0.53 (−1.2 to 0.12)	0.02 (−0.38 to 0.41)	−0.33 (−0.99 to 0.32)
≤5th percentile^c^	14.3	14.5	23.3	0.1	0.88 (0.61–1.28)	1.36 (0.77–2.38)	0.87 (0.59–1.29)	1.2 (0.68–2.13)
Behavioural difficulties, Total SDQ score								
mean (SD)	10.6 (5.9)	11.0 (6.1)	11.7 (5.9)	<0.001	0.31 (−0.25 to 0.86)	0.84 (−0.04 to 1.7)	0.28 (−0.27 to 0.84)	0.52 (−0.42 to 1.5)
≥90th percentile^c^	10.6	13.3	13.3	0.18	1.28 (0.93–1.75)	1.26 (0.80–1.99)	1.27 (0.93–1.75)	1.12 (0.69–1.81)
Visual disability, moderate or severe disabilities	1.0	1.0	2.0	0.006	1.11 (0.35–3.50)	2.27 (0.54–9.64)	1.07 (0.33–3.44)	2.08 (0.46–9.30)
Hearing disability, moderate or severe disabilities	0.8	1.4	2.3	0.1	1.49 (0.54–4.10)	2.59 (0.69–9.76)	1.39 (0.52–3.73)	1.29 (0.31–5.40)

Results after multiple imputation.

Percentages are weighted to consider the differences in survey design between gestational age groups.

aOR = adjusted odd ratios; CI = confidence interval; SD = Standard Deviation; GMFCS = Gross Motor Function Classification System; dB = decibel; FSIQ = Full scale intelligence quotient; MABC-2 = Movement Assessment Battery for Children- Second Edition; SDQ = Strengths and difficulties questionnaire; GA = Gestational Age.

a Model 1: adjusted for gestational age in week, maternal age at birth, mother birth in France, parents’ socio-economic status, cause of preterm birth, antenatal corticosteroids, magnesium sulphate administration, multiple pregnancy, sex, small-for-GA, Apgar at 5 min < 7, birth in type 3 and neonatal unit volume. Model 2 is model 1 variables and severe neonatal morbidities (late onset sepsis, severe cerebral lesion, severe bronchopulmonary dysplasia, necrotizing enterocolitis, severe retinopathy of prematurity).

b Severe = cerebral palsy gross motor function classification system (GMFCS) level 4/5, and/or bilateral binocular visual acuity <1/10, and/or uni or bilateral hearing loss >70 dB, and/or FSIQ < −3 SD; Moderate = cerebral palsy GMFCS level 2/3, and/or 3.2/10 < bilateral binocular visual acuity ≥1/10, and/or uni or bilateral hearing loss 40–70 dB and/or FSIQ between −3 and −2 SD; Mild = cerebral palsy GMFCS level 1, and/or 5/10 < uni or bilateral binocular visual acuity ≥3.2/10, and/or uni or bilateral hearing loss <40 db, and/or FSIQ between −2 and −1 SD, and/or total MABC-2 score ≤ 5th percentile, and/or behavioural difficulties according to SDQ score ≥90th percentile. Cut-off of the distribution related to a reference group born at term.^1^^,^^19^

c Cut-off of the distribution related to a reference group born at term.^1^^,^^19^

d Among children without cerebral palsy, severe or moderate sensory disabilities, and with full-scale intelligence quotient upper or equal than 2 standard deviations.

#### Secondary outcomes

Mild NDD occurred in 38.4%, 40.6%, and 42.1% of the unexposed, ≤7 days and >7 days groups, respectively (Table 2). Following adjustment for GA and the aforementioned confounders (model 1), there was a significant association between exposure for >7 days and the occurrence of mild NDD (Table 2). However, this association was no longer significant after additional adjustment for severe neonatal morbidities (model 2) (Table 2). The mean (standard deviation) FSIQ decreased according to the exposure status: 94.4 (15.3), 94.0 (15.9), and 87.6 (16.6) in the unexposed, ≤7 days and >7 days groups, respectively (Table 2). Exposure >7 days was associated with a negative adjusted mean difference (95% CI) in the FSIQ in model 1 but not in model 2 (Table 2 and Supplementary Table S4).

There were no significant differences between groups for the other secondary outcomes (CP, MABC-2 and SDQ scores, hearing, or visual impairment) (Table 2 and Supplementary Table S4).

#### Subgroup analyses

- *Neurodevelopment outcome by drug exposure:*

Following adjustment for GA and the aforementioned confounders (model 1), there were significant associations with moderate/severe NDD, and a lower FSIQ for children exposed to opioids with midazolam for more than seven days, but not for children exposed to opioids only (Table 3). After additional adjustment for severe neonatal morbidities (model 2), these associations were no longer significant (Table 3). As exposure to midazolam only was low, we did not perform an analysis for this drug.

- *Neurodevelopment outcome by sex*

**Table 3:** Neurodevelopmental outcome at five years according to exposure and duration of exposure, by drug.

	Unexposed, %	Exposed ≤7 days, %	Exposed >7 days, %	p Value	Model 1: aOR or adjusted mean difference (95% CI), vs Unexposed^a^		Model 2: aOR or adjusted mean difference (95% CI), vs Unexposed^a^	
					Exposed ≤7 days	Exposed >7 days	Exposed ≤7 days	Exposed >7 days
Opioids	n = 1952	n = 453	n = 113					
Neurodevelopmental disabilities^b^								
None	43.8	42.5	25.8	0.006	1 [Reference]	1 [Reference]	1 [Reference]	1 [Reference]
Mild	38.4	39.3	41.9		0.99 (0.74–1.32)	1.45 (0.72–2.93)	0.98 (0.73–1.30)	1.39 (0.71–2.72)
Moderate or Severe	17.8	18.1	32.3		0.97 (0.62–1.29)	1.91 (0.86–4.38)	0.91 (0.61–1.34)	1.40 (0.62–3.15)
Cerebral palsy	5.3	6.0	10.0	0.28	0.92 (0.58–1.46)	1.12 (0.48–2.63)	0.85 (0.50–1.45)	0.73 (0.29–1.83)
Full scale intelligence quotient (FSIQ)								
Mean (SD)	94.4 (15.3)	94.3 (15.8)	88.3 (16.0)	0.016	0.4 (−1.5 to 2.2)	−3.0 (−7.1 to 1.1)	0.4 (−1.5 to 2.2)	−1.4 (−5.3 to 2.5)
<−2 SD^c^	15.0	14.8	27.5	0.014	0.92 (0.63–1.34)	1.52 (0.82–2.81)	0.91 (0.62–1.33)	1.07 (0.55–2.08)
Developmental coordination disorders, MABC-2 score^d^								
Mean (SD)	9.5 (3.8)	9.5 (3.8)	8.4 (3.8)	0.17	0.1 (−0.4 to 0.6)	−0.5 (−1.5 to 0.6)	0.1 (−0.4 to 0.6)	−0.2 (−1.3 to 0.8)
≤5th percentile^c^	14.3	13.0	25.4	0.089	0.69 (0.36–1.29)	1.45 (0.67–3.16)	0.69 (0.37–1.30)	1.31 (0.58–2.97)
Behavioural difficulties, total SDQ score								
mean (SD)	10.6 (5.9)	10.8 (6.1)	11.6 (6)	0.26	0.0 (−0.6 to 0.7)	0.4 (−1.2 to 2.1)	0.0 (−0.6 to 0.7)	0.2 (−1.4 to 1.8)
≥90th percentile^c^	10.6	12.6	12.8	0.48	1.22 (0.86–1.73)	1.07 (0.52–2.18)	1.22 (0.86–1.73)	0.97 (0.47–2.00)
Opioids and midazolam	n = 1952	n = 218	n = 278					
Neurodevelopmental disabilities^b^								
None	43.8	35.3	26.9	<0.001	1 [Reference]	1 [Reference]	1 [Reference]	1 [Reference]
Mild	38.4	43.4	41.2		1.26 (0.86–1.85)	1.39 (0.96–2.02)	1.25 (0.85–1.83)	1.27 (0.83–1.94)
Moderate or Severe	17.8	21.3	31.9		1.19 (0.74–1.92)	2.10 (1.29–3.44)	1.04 (0.63–1.72)	1.36 (0.79–2.34)
Cerebral palsy	5.3	7.7	12	<0.001	1.13 (0.60–2.16)	1.50 (0.89–2.51)	0.88 (0.44–1.73)	0.80 (0.43–1.51)
Full scale intelligence quotient (FSIQ)								
Mean (SD)	94.4 (15.3)	92.8 (16.2)	87.5 (16.9)	<0.001	−0.01 (−2.6 to 2.6)	−4.5 (−7.2 to −1.7)	0.53 (−2.1 to 3.1)	−2.4 (−5.4 to 0.51)
<−2 SD^c^	15.0	17.1	27.4	<0.001	0.94 (0.59–1.51)	1.67 (1.04–2.69)	0.84 (0.52–1.37)	1.17 (0.68–2.02)
Developmental coordination disorders, MABC-2 score^d^								
Mean (SD)	9.5 (3.8)	9.0 (3.9)	8.4 (3.6)	0.008	−0.24 (−0.89 to 0.42)	−0.46 (−1.2 to 0.30)	−0.16 (−0.83 to 0.50)	−0.19 (−0.96 to 0.58)
≤5th percentile^c^	14.4	18.6	22.0	0.11	1.26 (0.72–2.20)	1.18 (0.61–2.28)	1.20 (0.68–2.11)	0.97 (0.48–1.98)
Behavioural difficulties, Total SDQ score								
Mean (SD)	10.6 (5.9)	11.6 (6.1)	11.9 (5.8)	0.002	0.70 (−0.23 to 1.6)	1.3 (0.32–2.2)	0.68 (−0.26 to 1.6)	1.0 (0.01–2.1)
≥90th percentile^c^	10.6	14.8	13.9	0.15	1.36 (0.82–2.24)	1.34 (0.80–2.24)	1.35 (0.81–2.24)	1.25 (0.71–2.19)

Results after multiple imputation.

Percentages are weighted to consider the differences in survey design between gestational age groups.

aOR = adjusted odds ratios; CI = confidence interval; SD = Standard Deviation; GMFCS = Gross Motor Function Classification System; dB = decibel; FSIQ = Full scale intelligence quotient; MABC-2 = Movement Assessment Battery for Children- Second Edition; SDQ = Strengths and difficulties questionnaire; GA = Gestational Age.

a Model 1: adjusted for gestational age in week, maternal age at birth, mother birth in France, parents’ socio-economic status, cause of preterm birth, antenatal corticosteroids, magnesium sulphate administration, multiple pregnancy, sex, small-for-GA, Apgar at 5 min < 7, birth in type 3 and neonatal unit volume. Model 2 is model 1 variables and severe neonatal morbidities (late onset sepsis, severe cerebral lesion, severe bronchopulmonary dysplasia, necrotizing enterocolitis, severe retinopathy of prematurity).

b Severe = cerebral palsy gross motor function classification system (GMFCS) level 4/5, and/or bilateral binocular visual acuity <1/10, and/or uni or bilateral hearing loss >70 dB, and/or FSIQ < −3 SD; Moderate = cerebral palsy GMFCS level 2/3, and/or 3.2/10 < bilateral binocular visual acuity ≥1/10, and/or uni or bilateral hearing loss 40–70 dB and/or FSIQ between −3 and −2 SD; Mild = cerebral palsy GMFCS level 1, and/or 5/10 < uni or bilateral binocular visual acuity ≥3.2/10, and/or uni or bilateral hearing loss <40 db, and/or FSIQ between −2 and −1 SD, and/or total MABC-2 score ≤ 5th percentile, and/or behavioural difficulties according to SDQ score ≥90th percentile. Cut-off of the distribution related to a reference group born at term.^1^^,^^19^

c Cut-off of the distribution related to a reference group born at term.^1^^,^^19^

d Among children without cerebral palsy, severe or moderate sensory disabilities, and with full-scale intelligence quotient upper or equal than 2 standard deviations.

Moderate/severe NDD occurred in 19.9%, 19.9%, and 34.8% in male children and in 15.7%, 17.7% and 28.2% in female children, in the unexposed, ≤7 days and >7 days groups, respectively (Supplementary Table S5). After adjustment for GA and the aforementioned confounders (model 1), there was an association between exposure >7 days and the occurrence of moderate/severe NDD in male and female, but more so among male than female (Supplementary Table S5). Following additional adjustment for severe neonatal morbidities (model 2), there was no longer any association between exposure and moderate/severe NDD at five years whatever the sex (Supplementary Table S5). A significantly lower FSIQ after more than seven days of exposure was observed only for males in model 1 (Supplementary Table S5).

- *Neurodevelopment outcome by GA group:*

Exposure >7 days was associated with more moderate/severe NDD, and a significant lower FSIQ in the very preterm group. In the extremely preterm group, exposure >7 days was associated with a significant lower FSIQ only (Supplementary Table S6). After additional adjustment for severe neonatal morbidities (model 2), there was no longer any significant association whatever the GA category (Supplementary Table S6).

### Sensitivity analyses

The results from the complete cases were in line with those of the main analysis with multiple imputations (Supplementary Table S4).

## Discussion

In this French prospective population-based cohort study of children born extremely and very preterm, the exposure rate to continuous opioids with/without midazolam was around 40%, but 62.7% in those born extremely preterm, with a wide variability between units. At five years, children exposed for more than seven days were more likely to have mild, or moderate/severe NDD compared to unexposed children. This occurrence of moderate/severe NDD was mainly associated with lower FSIQs rather than with motor, behavioural or sensory impairment. However, after adjustment for severe neonatal morbidities, no further associations were observed between neonatal exposure and neurodevelopment at five years.

This study has several strengths. To our knowledge, this cohort is the largest to investigate the potential role of exposure to opioids with/without midazolam on neurodevelopmental outcomes at five years among children born extremely and very preterm. The large size of the cohort allowed two groups of children born extremely and very preterm to be studied, as well as those exposed to opioids with/without midazolam. The results of the sensitivity analyses were consistent with those of the primary analysis, providing further confidence in the reliability of the results. Another strength is the population-based design, at a national level, with prospective enrolment, and the use of standardised tools calibrated using a contemporary cohort of children born at term to assess development in several developmental dimensions.^1^^,^^23^ This study also has limitations. EPIPAGE-2 was not specifically designed to assess the effect of opioids with/without midazolam on neurodevelopment at five years, and data that could have been useful are missing. The reason for exposure was not recorded, nor were the doses used for each drug. This could have provided additional information, as a possible cumulative toxicity has been suggested for morphine,^11^^,^^14^^,^^16^ and duration is not always associated with higher doses. The sequence of opioid administration when different opioids were administered was also not recorded (e.g., sufentanil and morphine, morphine and fentanyl), and this did not allow us to assess neurodevelopmental outcomes based on the type of opioid used. However, the use of exposure duration ≤7 days or >7 days allows comparison with other studies. Information on pain and pain relief, including parental presence and non-pharmacological approaches, was not available. Both exposure to opioids with/without midazolam and to pain may affect long-term neurodevelopment.^4^, ^5^, ^6^ Pain management can be highly variable between units,^8^, ^9^, ^10^^,^^18^ and this was residual confounding that we were not able to control for. EPIPAGE-2 study collected only severe IVH and cPVL as severe brain lesions, but not other lesions affecting the developing brain, such as large cerebellar lesions or large deep grey matter lesions, that may also be associated with worse outcome. Finally, the attrition rate over time was high, but this was mitigated by the use of multiple imputation, and the comparable rate of exposure between participating and non-participating children limited the selection bias. This may have underestimated the rates of NDD but not the direction of associations.

In a previous analysis of the EPIPAGE-2 study, survival was higher in the exposed group with no observed difference in sensorimotor outcome at two years corrected age after continuous opioid and/or midazolam infusion in ventilated very preterm neonates.^24^ Exposure was however defined differently and focused on the period of initial mechanical ventilation, the number of extremely preterm neonates, at high risk of NDD, was lower, and the median duration of first exposure was four days.^24^ In our study, the five-year survival rate was higher in the unexposed children. However, this was due to a higher in-hospital mortality rate in both exposed groups, as mortality after hospital discharge was low in all three groups. The in-hospital mortality rates differed between the three groups, as did the median age at death and the causes of death. These results are in line with the differences in baseline characteristics since the most premature infants, who are at highest risk of death, were also more frequently exposed. We assumed that exposure to opioids with/without midazolam was not the direct cause of death; rather, it was a proxy for the occurrence of severe neonatal morbidities that motivated the use of these drugs. The results of this study should be interpreted in the light of survival, and the association between exposure >7 days and moderate/severe NDD is likely to be underestimated, as children who did not survive would also have been at a higher risk of NDD.

The sickest neonates are likely to be most exposed, opioids and sedatives may be cause or consequence of neonatal morbidity, and neonatal morbidity is strongly associated with neurodevelopmental outcomes.^4^, ^5^, ^6^ The role of opioids *per se* is therefore difficult to explore, and “opioids and/or midazolam are not solely to be blamed”.^25^ In the secondary analysis of the Preterm Erythropoietin Neuroprotection (PENUT) trial, the decision was made not to adjust for neonatal morbidity “as events largely occur after opioids exposure and would not be confounders”.^8^ However, depending on the clinical situation, opioids may be considered as confounders or mediators. Indeed, prolonged use of opioids may prolong mechanical ventilation and possible subsequent BDP or intestinal hypomotility and subsequent NEC. However, NEC can also occur suddenly and require opioid treatment to relieve the induced pain. The data available in EPIPAGE-2 were not precise enough to describe the different situations. Furthermore, the temporality and causes of exposure were not available. Despite these limitations, we elected to present a model adjusted for neonatal morbidities, given that neonates who are the sickest and have the greatest number of neonatal morbidities, influencing the occurrence of NDD, are also the ones who are exposed the longest. A propensity score-matched analysis might have been more appropriate, but the number of neonates born at 24–27 weeks and exposed to opioids with/without midazolam was too large to obtain good matching. Finally, after adjustment for severe neonatal morbidities, there was no longer an association between prolonged exposure and later neurological development, also highlighting the important role of these factors in later neurodevelopment. Future research should accurately record the timing of exposure to opioids and/or sedatives, the occurrence of neonatal morbidities, and also the cumulative doses of drugs and the exposure to pain and pain relief to provide new insights into this question.

Our results support those observed at 18–24 months in children born extremely or very preterm, showing an association between exposure of more than seven days and poorer developmental outcome, with exposure to opioids and benzodiazepines being the most detrimental.^7^^,^^8^^,^^26^ At five years, the first EPIPAGE study reported that prolonged exposure to sedation and/or analgesia was not associated with moderate or severe NDD in very preterm neonates.^15^ This study was conducted in 1997, and only 7% of the total population was exposed to these drugs, a sample size insufficient to provide reliable conclusions.^15^ In children born in the 2000s, Luzzati et al. reported lower FSIQs at five years in 106 children born extremely preterm and exposed to a higher cumulative dose of morphine.^14^ In another study,^16^ in a group of 47 children born very preterm, an increase in cumulative opiate exposure did not alter cognitive and motor outcomes, but was associated with an increase in autism spectrum disorders and withdrawn behaviour at five years.^16^ The large size of our cohort and the use of opioids with/without midazolam allow for results that appear more robust. Neurotoxicity may be related to the drug itself, as morphine impairs cerebellar maturation,^11^ and midazolam alters hippocampal growth,^12^ but also to cumulative exposure with additive toxicity.^4^^,^^10^, ^11^, ^12^ In case of association, the increased risk may be the result of different effects of the drugs on the developing brain.^11^^,^^12^ Sex is also a factor to consider when analysing pain management practices and long-term neurodevelopmental outcomes in children born preterm. Indeed, if females appear to be more susceptible to procedural pain, with associated altered maturation on neonatal structural connectivity,^27^ males appear to be more susceptible to midazolam exposure, associated with impaired hippocampal growth, with long lasting effects at school age.^26^ Our study highlights the vulnerability of male children, compared to female, after an exposure for more than seven days. This may be due to the male sex, which is a known associated factor for NDD after a preterm birth,^1^ but also to a higher vulnerability of exposure to midazolam during the neonatal period.^26^ We also showed that the lower FSIQ associated with a prolonged exposure to opioids with/without midazolam increased the rate of moderate/severe NDD but also the rate of mild NDD, especially among very preterm but not extremely preterm neonates. This may be attributed to the higher frequency of exposure among extremely preterm neonates compared to very preterm neonates, as well as the smaller number of surviving extremely preterm neonates in our cohort. This is an important observation from a public health perspective, because the overall rate of mild NDD is always higher than the rate of moderate/severe NDD, which was approximately 40% in the EPIPAGE-2 cohort.^1^ We did not observe an association between exposure and an increased rate of behavioural problems. The SDQ score we used is designed to screen for behavioural difficulties.^1^^,^^28^ The use of a test designed to assess social communication difficulties may have more accurately identified withdrawn behaviour and the risk of autism. Further research should specifically assess the risk of autism spectrum disorders, as genetic factors may also be involved in subsequent neurodevelopment.^6^^,^^29^^,^^30^

Finally, our results confirm the previously described considerable variability in exposure to opioids with/without midazolam between NICUs.^8^, ^9^, ^10^ It is interesting to note that the NICUs where the neonates were most exposed had the lowest volume of activity, which may be related to systematic practices rather than individualised care. As exposure to opioids with/without midazolam is a modifiable behaviour, and given the concern on adverse long-term neurodevelopmental effects of such exposure, recommendations with standardised evidence-based protocols for the management of acute or prolonged pain and distress in these high-risk neonates need to be developed.^9^

### Conclusion

The incidence of exposure to opioids and/or benzodiazepines in very preterm neonates throughout the NICUs is high. Completely avoiding strong pharmacological analgesia or sedation in this population is neither feasible nor acceptable in clinical practice. However, exposure of more than seven days, mainly to a combination opioids-midazolam, might be associated with cognitive impairment at preschool age, with severe morbidities being a major intermediate factor acting as cause or consequence of exposure to these drugs. Further clinical trials of opioids and/or sedatives such as dexmedetomidine for pain and stress management in preterm neonates are warranted. Drug-saving techniques such as non-pharmacological methods and family-centred care should be widely implemented.

## Contributors

Dr Walter-Nicolet, Dr Pierrat, Dr Durrmeyer, Dr Ancel and Mrs Marchand-Martin had full access to all the data in the study and take responsibility for the integrity of the data and the accuracy of the data analysis. Study concept and design were done by Dr Walter-Nicolet, Dr Pierrat, Dr Durrmeyer, Dr Morgan. Acquisition, analysis, or interpretation of data were done by Mrs Marchand-Martin, Dr Walter-Nicolet, Dr Pierrat, Dr Durrmeyer and Dr Morgan. Drafting of the manuscript was done by Dr Walter-Nicolet. Critical revision of the manuscript for important intellectual content was done by all authors. Dr Ancel and Mrs Benhammou obtained funding (EPIPAGE-2). Dr Pierrat, Dr Durrmeyer supervised the study and had final responsibility for the decision to submit for publication.

## Data sharing statement

The procedures carried out with the French data privacy authority (CNIL, Commission Nationale de l’Informatique et des Libertés) do not provide for the transmission of the database. Nevertheless, consultation by the editorial board or interested researchers may be considered, subject to prior determination of the terms and conditions of such consultation and in respect for compliance with the applicable regulations.

Data access procedures can be found on the EPIPAGE-2 website [https://epipage2.inserm.fr/index.php/en/related-research/access-to-epipage-2-data].

## Declaration of generative AI and AI-assisted technologies in the writing process

During the preparation of this work the authors used the DeepL Write tool (https://www.deepl.com/en/write) in order to improve the readability and English of the draft. After using this tool, the author(s) reviewed and edited the content as needed and take full responsibility for the content of the publication.

The final version of the text was reviewed by Dr Morgan, a native English-speaker.

## Declaration of interests

For this study, Dr Elizabeth Walter-Nicolet received a financial contribution from the Apicil Foundation (Fondation Apicil, Lyon, France) to carry out a PhD.

All authors have nothing to disclose.

## Appendix ASupplementary data

Supplementary Fig. S1 and Tables S1–S6

Translated abstract
