# Supplementary material for: Neurodevelopmental outcomes at five years in children born very preterm (24–31 weeks) exposed to opioids with or without midazolam: results from the French nationwide EPIPAGE-2 cohort study
Source: Lancet Reg Health Eur. 2025 Feb 18;52:101242. doi: 10.1016/j.lanepe.2025.101242 (PMC11883393; doi:10.1016/j.lanepe.2025.101242)
Supplement: Supplementary Fig. S1 and Tables S1–S6 [file mmc1.pdf]

Supplemental material

Neurodevelopmental outcomes at five years in children born very preterm (24-31 weeks) exposed to opioids with or without midazolam: results from the French nationwide EPIPAGE-2 cohort study.

Table of contents

Supplementary Table 1. Maternal and neonatal characteristics of participating and non-participating children at five years. 2

Supplementary Table 2. Deaths in overall cohort, N=3510. 3

Supplementary Table 3. Distribution of opioid prescriptions by type of drug among children exposed. 4

Supplementary Table 4. Neurodevelopmental outcome at five years by exposure and duration of exposure to opioids with/without midazolam. Complete cases. 5

Supplementary Table 5. Neurodevelopmental outcome at five years according to exposure and duration of exposure to opioids with/without midazolam, by sex. Results after multiple imputation. ... 6

Supplementary Table 6. Neurodevelopmental outcome at five years according to exposure and duration of exposure to opioids with/without midazolam, by gestational age group. Results after multiple imputation. 8

Supplementary Figure 1. 10

Supplementary references 11

Supplementary Table 1. Maternal and neonatal characteristics of participating and non-participating children at five years.

	Included population analysis with complete assessment at five years (n=1883)		Non-participating (n=897) or incomplete assessment (n=337) at five years		
	n/N	%	n/N	%	P Value
**Maternal characteristics at birth**					
**Maternal age**					
<25 years	283/1883	15·0	302/1234	24·5	<0·001
25-34 years	1189/1883	63·1	663/1234	53·7	
≥35 years	411/1883	21·9	269/1234	21·8	
**Birth in France**	1503/1876	80·2	847/1206	70·4	<0·001
**Parents' socio-economic status***					
Executive	453/1804	25·0	179/1144	15·7	<0·001
Intermediate	436/1804	24·3	168/1144	14·8	
Administration	469/1804	26·3	338/1144	29·6	
Service, trade	227/1804	12·4	201/1144	17·5	
Worker, unemployed	219/1804	12·1	258/1144	22·4	
**Maternal obstetrical characteristics**					
**Multiple pregnancy**	629/1883	33·4	383/1234	31·1	0·19
**Cause of preterm birth**					
Preterm labour group	740/1883	38·7	477/1234	38·3	0·40
Preterm premature rupture of membranes	446/1883	23·4	301/1234	24	
Hypertensive disorders or Placental abruption group	444/1883	24·1	271/1234	22·5	
Isolated foetal growth restriction	119/1883	6·5	73/1234	6·2	
Other	134/1883	7·2	112/1234	9	
**Antenatal corticosteroids**	1582/1850	85·6	1011/1212	83·4	0·11
**Magnesium sulphate administration**	177/1858	9·6	90/1211	7·5	0·052
**Neonatal characteristics**					
**Gestational age groups (weeks)**					
24	31/1883	1·3	21/1234	1·3	0·46
25	97/1883	4·0	77/1234	4·8	
26	178/1883	7·3	107/1234	6·7	
27	196/1883	10·9	114/1234	9·7	
28	246/1883	13·6	146/1234	12·4	
29	279/1883	15·5	190/1234	16·1	
30	387/1883	21·4	240/1234	20·3	
31	469/1883	26	339/1234	28·7	
**Sex, male**	968/1883	51·5	630/1234	51·1	0·85
**Small for gestational age†**	673/1883	36·4	422/1234	34·8	0·37
**Apgar at 5min <7**	299/1800	16·2	187/1158	15·6	0·67
**Severe neonatal morbidities**					
Severe cerebral abnormalities	94/1858	4·9	69/1221	5·3	0·59
Necrotizing enterocolitis	67/1862	3·5	42/1224	3·4	0·79
Severe bronchopulmonary dysplasia	150/1837	7·5	88/1193	6·6	0·34
Severe retinopathy of prematurity	24/1873	1·1	15/1219	1·0	0·76
Late onset sepsis	418/1860	21·2	243/1218	18·8	0·1
**Treated with opioids and/or midazolam**					
Untreated	1183/1883	64·2	769/1234	63·9	0·77
Treated ≤ 7 days	454/1883	24·0	308/1234	24·9	
Treated > 7 days	246/1883	11·7	157/1234	11·1	
**Units' characteristics**					
**Birth in type 3**	1643/1883	87·1	1052/1234	85·2	0·22
**Neonatal unit volume activity‡**					
<55	674/1883	36·1	464/1234	38·0	0·66
[55-70[	407/1883	21·6	270/1234	21·8	
[70-90[	317/1883	16·9	202/1234	16·4	
>=90	485/1883	25·5	298/1234	23·8	

Data are No./total (%), unless otherwise indicated. Denominators vary according to the number of missing data for each variable. Percentages are weighted to consider the differences in survey design between gestational age groups, resulting in numbers different from No./total calculation. GA=gestational age.

* Defined as the highest occupational status between occupations of the mother and the father, or mother only if living alone.

† Small for GA was defined as birth weight less than the 10th percentile for gestational age and sex based on French intrauterine "EPOPé" growth curves.[1]

‡ Number of neonates born before 32 GA admitted in 2011, obtained from the national hospital discharge database.

Supplementary Table 2. Deaths in overall cohort, N=3510.

	Unexposed (n=2017)		Exposed ≤ 7 days (n=956)		Exposed >7 days (n=537)		
	**n/N**	**%**	**n/N**	**%**	**n/N**	**%**	**P value**
**Deaths in the NICU**	55/2017	2·3	190/956	18·0	129/537	23·7	<0·001
**Deaths between discharge and 5 years**	10/2017	0·5	4/956	0·4	5/537	1·0	
**Survivors at five years**	1952/2017	97·2	762/956	81·6	403/537	75·3	
**Deaths in the NICU**							
**Age at death (day),** median [IQR]	55	3 [1-9]	190	5 [2-9]	129	19 [13-32]	
**Cause of death in the NICU**							
Respiratory distress syndrome	27/55	47·8	67/190	34·6	38/129	29·9	0·046
NEC	1/55	2·2	12/190	7·0	8/129	6·4	
Infection	5/55	8·6	19/190	10·2	20/129	15·2	
CNS Injury	5/55	8·0	55/190	27·9	38/129	28·9	
Other	10/55	19·4	21/190	11·6	19/129	14·9	
Unknown	5/55	9·7	11/190	5·6	5/129	3·9	
Congenital anomaly	2/55	4·3	5/190	3·1	1/129	0·7	

Percent are weighted to consider the differences in survey design between gestational age groups. NICU=neonatal intensive care unit, NEC=necrotizing enterocolitis, CNS=central nervous system

Supplementary Table 3. Distribution of opioid prescriptions by type of drug among children exposed.

	Exposed to opioids only or both opioids + midazolam n=1062 (%)	Exposed to opioids only n=566 (%)	Exposed to both opioids + midazolam n=496 (%)
Sufentanil only	526/1062 (49·5)	264/566 (46·6)	262/496 (52·8)
Morphine only	200/1062 (18·8)	145/566 (25·6)	55/496 (11·1)
Fentanyl only	142/1062 (13·4)	94/566 (16·6)	48/496 (9·7)
Sufentanil - Morphine	153/1062 (14·4)	47/566 (8·3)	106/496 (21·4)
Fentanyl - Morphine	32/1062 (3·0)	15/566 (2·7)	17/496 (3·4)
Sufentanil - Fentanyl	3/1062 (0·3)	1/566 (0·2)	2/496 (0·4)
Sufentanil – Morphine - Fentanyl	6/1062 (0·6)	0/566 (0·0)	6/496 (1·2)

The median (Inter Quartile Rate) age at start and duration of exposure were: 1 (0-8) day during 3 (1-7) days for sufentanil; 3 (0-21) days during 8 (3-19) days for morphine, and 1 (0-3) day during 2 (1-6) days for fentanyl.

Supplementary Table 4. Neurodevelopmental outcome at five years by exposure and duration of exposure to opioids with/without midazolam. Complete cases.

	Unexposed (n=1952), %	Exposed ≤ 7 days (n=762), %	Exposed >7 days (n=403), %	P Value	Model 1: aOR or adjusted mean difference (95% CI), vs Unexposed*		Model 2: aOR or adjusted mean difference (95% CI), vs Unexposed*	
					Exposed ≤ 7 days	Exposed > 7 days	Exposed ≤ 7 days	Exposed > 7 days
**Neurodevelopmental disabilities***	n=1183	n=454	n=246	<0·001				
None	52·2	48·1	31·5		1 [Reference]	1 [Reference]	1 [Reference]	1 [Reference]
Mild	34.0	38·3	42·1		1·02 (0·77 to 1·35)	1·35 (0·88 to 2·08)	1·02 (0·76 to 1·37)	1·33 (0·83 to 2·15)
Moderate or Severe	13·8	13·5	26·4		1·00 (0·69 to 1·46)	2·16 (1·25 to 3·72)	0·89 (0·64 to 1·24)	1·37 (0·74 to 2·54)
**Cerebral palsy**	4·9	5.0	11·2	<0·001	0·88 (0·54 to 1·45)	1·55 (0·89 to 2·67)	0·77 (0·45 to 1·29)	0·76 (0·41 to 1·40)
**Full scale intelligence quotient (FSIQ)**								
mean (SD)	96·7 (14·6)	96·2 (15·5)	90·0 (16·5)	<0·001	-0·20 (-2·0 to 1·6)	-3·7 (-6·3 to -1·1)	0·02 (-1·7 to 1·7)	-1·9 (-4·5 to 0·70)
< -2 SD†	11·0	10·4	20·6	<0·001	1·06 (0·69 to 1·61)	1·89 (1·12 to 3·20)	0·98 (0·67 to 1·42)	1·23 (0·69 to 2·17)
**Developmental Coordination Disorders, MABC-2 score**								
mean (SD)	10·2 (3·1)	9·8 (3·1)	8·8 (3·1)	<0·001	-0·09 (-0·48 to 0·30)	-0·57 (-1·2 to 0·07)	-0·01 (-0·42 to 0·39)	-0·33 (-1·0 to 0·35)
≤ 5th percentile †,‡	7·2	7·6	18·3	<0·001	0·80 (0·52 to 1·23)	1·63 (0·83 to 3·21)	0·81 (0·50 to 1·29)	1·53 (0·72 to 3·27)
**Developmental Coordination Disorders, MABC-2 score**								
mean (SD)	10·2 (3·1)	9·8 (3·1)	8·8 (3·1)	<0·001	-0·09 (-0·48 to 0·30)	-0·57 (-1·2 to 0·07)	-0·01 (-0·42 to 0·39)	-0·33 (-1·0 to 0·35)
≤ 5th percentile †,‡	7·2	7·6	18·3	<0·001	0·80 (0·52 to 1·23)	1·63 (0·83 to 3·21)	0·81 (0·50 to 1·29)	1·53 (0·72 to 3·27)
**Behavioural difficulties, Total SDQ score**								
mean (SD)	10·3 (5·8)	10·7 (6·1)	11·4 (5·7)	0·014	0·14 (-0·50 to 0·79)	0·80 (-0·19 to 1·8)	0·11 (-0·58 to 0·80)	0·30 (-0·76 to 1·4)
≥ 90th percentile †	9·3	12·8	11·4	0·090	1·41 (0·98 to 2·03)	1·23 (0·75 to 2·03)	1·46 (0·99 to 2·16)	1·00 (0·56 to 1·78)
**Visual disability,** moderate or severe disabilities	0·4	0·5	1·6	0·095	-	-	-	-
**Hearing disability,** moderate or severe disabilities	0·5	1·2	2·2	0·016	-	-	-	-

Percentages are weighted to consider the differences in survey design between gestational age groups. aOR=adjusted odd ratios; CI=confidence interval; SD=Standard Deviation; GMFCS=Gross Motor Function Classification System; dB=decibel; FSIQ=Full scale intelligence quotient; MABC-2=Movement Assessment Battery for Children- Second Edition; SDQ=Strengths and difficulties questionnaire; GA=Gestational Age.

* Model 1: adjusted for gestational age in week, maternal age at birth, mother birth in France, parents' socio-economic status, cause of preterm birth, antenatal corticosteroids, magnesium sulphate administration, multiple pregnancy, sex, small-for-GA, Apgar at 5 min < 7, birth in type 3 and neonatal unit volume.
Model 2 is model 1 variables and severe neonatal morbidities (late onset sepsis, severe cerebral lesion, severe bronchopulmonary dysplasia, necrotizing enterocolitis, severe retinopathy of prematurity).

† Severe=cerebral palsy gross motor function classification system (GMFCS) level 4/5, and/or bilateral binocular visual acuity < 1/10, and/or uni or bilateral hearing loss > 70 dB, and/or FSIQ < -3 SD; Moderate=cerebral palsy GMFCS level 2/3, and/or 3.2/10 < bilateral binocular visual acuity ≥ 1/10, and/or uni or bilateral hearing loss 40 to 70 dB and/or FSIQ between -3 to-2 SD; Mild=cerebral palsy GMFCS level 1, and/or 5/10 < uni or bilateral binocular visual acuity ≥ 3.2/10, and/or uni or bilateral hearing loss < 40 db, and/or FSIQ between -2 to-1 SD, and/or total MABC-2 score ≤ 5th percentile, and/or behavioural difficulties according to SDQ score ≥ 90th percentile. Cut-off of the distribution related to a reference group born at term.[2,3]
‡ Cut-off of the distribution related to a reference group born at term.[2,3]
§ Among children without cerebral palsy, severe or moderate sensory disabilities, and with full-scale intelligence quotient upper or equal than 2 standard deviations.

Supplementary Table 5. Neurodevelopmental outcome at five years according to exposure and duration of exposure to opioids with/without midazolam, by sex. Results after multiple imputation.

	Unexposed , %	Exposed ≤ 7 days, %	Exposed >7 days, %	P Value	Model 1: aOR or adjusted mean difference (95% CI), vs Unexposed*		Model 2: aOR or adjusted mean difference (95% CI), vs Unexposed*	
					Exposed ≤ 7 days	Exposed > 7 days	Exposed ≤ 7 days	Exposed > 7 days
**Male**	n= 986	n= 399	n= 213					
**Neurodevelopmental disabilities***								
None	41·2	38·6	21·7	<0·001	1 [Reference]	1 [Reference]	1 [Reference]	1 [Reference]
Mild	38·9	41·5	43·5		1·07 (0·77 to 1·46)	1·83 (1·04 to 3·23)	1·07 (0·78 to 1·48)	1·67 (0·78 to 3·57)
Moderate or Severe	19·9	19·9	34·8		0·93 (0·61 to 1·42)	2·35 (1·20 to 4·62)	0·89 (0·58 to 1·38)	1·79 (0·96 to 3·35)
**Cerebral palsy**	5·1	6·4	13·5	<0·001	0·97 (0·56 to 1·69)	1·54 (0·86 to 2·78)	0·78 (0·42 to 1·44)	1·85 (0·43 to 1·67)
**Full scale intelligence quotient (FSIQ)**								
mean (SD)	93·5 (15·5)	93·5 (16·0)	86·1 (16·5)	<0·001	0·62 (-1·5 to 2·7)	-4·8 (-7·9 to -1·6)	0·78 (-1·3 to 2·9)	-2·8 (-6·3 to 0·59)
< -2 SD†	16·9	16·6	30·5	<0·001	0·87 (0·57 to 1·35)	1·60 (0·92 to 2·77)	0·84 (0·54 to 1·32)	1·18 (0·63 to 2·22)
**Developmental Coordination Disorders, MABC-2 score**								
mean (SD)	9·2 (3·8)	9·0 (3·7)	7·8 (3·7)	0·003	0·00 (-0·52 to 0·51)	-0·78 (-1·7 to 0·14)	0·01 (-0·51 to 0·53)	-0·61 (-1·6 to 0·37)
≤ 5th percentile †,‡	16·3	16·1	30·1	0·005	0·89 (0·55 to 1·43)	1·80 (0·92 to 3·52)	0·88 (0·54 to 1·45)	1·66 (0·80 to 3·44)
**Behavioural difficulties, Total SDQ score**								
mean (SD)	11·5 (6·0)	11·7 (6·0)	12·5 (6·1)	0·082	0·17 (-0·67 to 1·0)	0·78 (-0·41 to 2·0)	0·15 (-0·70 to 1·0)	0·49 (-0·75 to 1·7)
≥ 90th percentile †	13·5	14·7	16·8	0·57	1·10 (0·73 to 1·66)	1·35 (0·72 to 2·52)	1·10 (0·73 to 1·66)	1·16 (0·60 to 2·26)
**Female**	n= 966	n= 363	n= 190					
**Neurodevelopmental disabilities***								
None	46·4	42·8	31·2	0·005	1 [Reference]	1 [Reference]	1 [Reference]	1 [Reference]
Mild	38·0	39·6	40·6		1·07 (0·75 to 1·52)	1·19 (0·71 to 1·98)	1·05 (0·74 to 1·49)	1·11 (0·67 to 1·86)
Moderate or Severe	15·7	17·7	28·2		1·12 (0·71 to 1·75)	1·95 (1·002 to 3·78)	1·03 (0·66 to 1·62)	1·29 (0·65 to 2·59)
**Cerebral palsy**	5·5	6·1	9·0	0·34	0·93 (0·52 to 1·66)	1·44 (0·68 to 3·06)	0·85 (0·45 to 1·59)	0·88 (0·36 to 2·15)
**Full scale intelligence quotient (FSIQ)**								
mean (SD)	95·4 (14·9)	94·5 (15·9)	89·3 (16·4)	<0·001	-0·36 (-2·6 to 1·9)	-3·4 (-6·9 to 0·05)	-0·05 (-2·3 to 2·2)	-1·7 (-5·2 to 1·9)
< -2 SD†	13·0	13·9	23·5	0·008	0·99 (0·62 to 1·58)	1·60 (0·86 to 2·96)	0·93 (0·58 to 1·50)	1·07 (0·53 to 2·17)
**Developmental Coordination Disorders, MABC-2 score**								
mean (SD)	9·9 (3·8)	9·8 (3·8)	9·0 (3·5)	0·091	-0·04 (-0·61 to 0·54)	-0·26 (-1·3 to 0·74)	-0·02 (-0·60 to 0·56)	-0·12 (-1·1 to 0·83)
≤ 5th percentile †,‡	12·4	12·7	16·5	0·36	0·84 (0·43 to 1·67)	0·95 (0·42 to 2·18)	0·84 0·42 to 1·69	0·87 0·38 to 2·02
**Behavioural difficulties, Total SDQ score**								
mean (SD)	9·8 (5·6)	10·3 (6·2)	10·8 (5·5)	0·039	0·53 (-0·31 to 1·4)	0·94 (-0·23 to 2·1)	0·49 (-0·35 to 1·3)	0·56 (-0·64 to 1·8)
≥ 90th percentile †	7·6	11·7	9·3	0·16	1·61 (0·92 to 2·83)	1·15 (0·57 to 2·32)	1·58 (0·90 to 2·78)	1·04 (0·50 to 2·17)

Percentages are weighted to consider the differences in survey design between gestational age groups. aOR=adjusted odd ratios; CI=confidence interval; SD=Standard Deviation; GMFCS=Gross Motor Function Classification System; dB=decibel; FSIQ=Full scale intelligence quotient; MABC-2=Movement Assessment Battery for Children- Second Edition; SDQ=Strengths and difficulties questionnaire; GA=Gestational Age.

* Model 1: adjusted for gestational age in week, maternal age at birth, mother birth in France, parents' socio-economic status, cause of preterm birth, antenatal corticosteroids, magnesium sulphate administration, multiple pregnancy, sex, small-for-GA, Apgar at 5 min < 7, birth in type 3 and neonatal unit volume.
Model 2 is model 1 variables and severe neonatal morbidities (late onset sepsis, severe cerebral lesion, severe bronchopulmonary dysplasia, necrotizing enterocolitis, severe retinopathy of prematurity).

† Severe=cerebral palsy gross motor function classification system (GMFCS) level 4/5, and/or bilateral binocular visual acuity < 1/10, and/or uni or bilateral hearing loss > 70 dB, and/or FSIQ < -3 SD; Moderate=cerebral palsy GMFCS level 2/3, and/or 3.2/10 < bilateral binocular visual acuity ≥ 1/10, and/or uni or bilateral hearing loss 40 to 70 dB and/or FSIQ between -3 to-2 SD; Mild=cerebral palsy GMFCS level 1, and/or 5/10 < uni or bilateral binocular visual acuity ≥ 3.2/10, and/or uni or bilateral hearing loss < 40 db, and/or FSIQ between -2 to-1 SD, and/or total MABC-2 score ≤ 5th percentile, and/or behavioural difficulties according to SDQ score ≥ 90th percentile. Cut-off of the distribution related to a reference group born at term.[2,3]

‡ Cut-off of the distribution related to a reference group born at term.[2,3]

§ Among children without cerebral palsy, severe or moderate sensory disabilities, and with full-scale intelligence quotient upper or equal than 2 standard deviations.

Supplementary Table 6. Neurodevelopmental outcome at five years according to exposure and duration of exposure to opioids with/without midazolam, by gestational age group. Results after multiple imputation.

	Unexposed, %	Exposed ≤ 7 days, %	Exposed >7 days, %	P Value	Model 1: aOR or adjusted mean difference (95% CI), vs Unexposed*		Model 2: aOR or adjusted mean difference (95% CI), vs Unexposed*	
					Exposed ≤ 7 days	Exposed > 7 days	Exposed ≤ 7 days	Exposed > 7 days
**24-27 weeks**	n=295	n=223	n=303					
**Neurodevelopmental disabilities†**								
None	30·9	34·8	24·8	0·13	1 [Reference]	1 [Reference]	1 [Reference]	1 [Reference]
Mild	43·6	40·9	42·1		0·86 (0·56 to 1·32)	1·23 (0·76 to 2·00)	0·87 (0·56 to 1·35)	1·20 (0·71 to 2·03)
Moderate or Severe	25·5	24·2	33·1		0·92 (0·53 to 1·60)	1·69 (0·93 to 3·07)	0·85 (0·47 to 1·51)	1·18 (0·64 to 2·20)
**Cerebral palsy**	10·9	9·5	12·7	0·56	0·87 (0·43 to 1·76)	1·18 (0·64 to 2·18)	0·67 (0·32 to 1·43)	0·72 (0·37 to 1·40)
**Full scale intelligence quotient (FSIQ)**								
mean (SD)	90·7 (15·6)	91·6 (17·1)	86·7 (15·9)	0·007	0·7 (-2·3 to 3·6)	-3·7 (-6·8 to -0·6)	0·9 (-2·0 to 3·9)	-2·1 (-5·2 to 1·0)
< -2 SD‡	20·9	20·4	28·8	0·084	0·98 (0·55 to 1·72)	1·43 (0·85 to 2·42)	0·92 (0·52 to 1·62)	1·06 (0·61 to 1·85)
**Developmental Coordination Disorders, MABC-2 score§**								
mean (SD)	9·2 (3·8)	8·7 (3·7)	8·1 (3·6)	0·013	-0·4 (-1·2 to 0·5)	-0·7 (-1·6 to 0·1)	-0·4 (-1·2 to 0·5)	-0·6 (-1·5 to 0·3)
≤ 5th percentile ‡	17·0	19·2	23·5	0·26	1·05 (0·54 to 2·06)	1·20 (0·63 to 2·29)	1·05 (0·52 to 2·10)	1·07 (0·54 to 2·11)
**Behavioural difficulties, Total SDQ score**								
mean (SD)	11·2 (6)	11·4 (5·9)	11·5 (5·9)	0·60	0·5 (-0·6 to 1·6)	0·6 (-0·5 to 1·8)	0·6 (-0·6 to 1·7)	0·5 (-0·7 to 1·7)
≥ 90th percentile‡	12·4	12	13·4	0·79	1·09 (0·59 to 2·02)	1·23 (0·64 to 2·37)	1·10 (0·59 to 2·04)	1·15 (0·59 to 2·24)
**28-31 weeks**	n=1657	n=539	n=100					
**Neurodevelopmental disabilities†**								
None	45·7	42·6	29·4	0·028	1 [Reference]	1 [Reference]	1 [Reference]	1 [Reference]
Mild	37·7	40·4	42·2		1·12 (0·72 to 1·56)	1·67 (0·92 to 3·04)	1·12 (0·84 to 1·48)	1·43 (0·79 to 2·57)
Moderate or Severe	16·6	17·0	28·4		1·06 (0·72 to 1·56)	2·68 (1·32 to 5·45)	1·02 (0·69 to 1·50)	1·90 (0·84 to 4·32)
**Cerebral palsy**	4·4	5·1	8·3	0·29	1·18 (0·66 to 2·10)	2·03 (0·89 to 4·66)	1·08 (0·58 to 2·00)	1·00 (0·38 to 2·61)
**Full scale intelligence quotient (FSIQ)**								
mean (SD)	95·0 (15·2)	94·8 (15·4)	89·8 (17·8)	0·084	-0·1 (-1·9 to 1·8)	-4·6 (-8·6 to -0·7)	0·1 (-1·7 to 2·0)	-2·6 (-6·8 to 1·6)
< -2 SD‡	14·0	13·5	23·3	0·085	0·94 (0·65 to 1·35)	1·93 (1·01 to 3·70)	0·91 (0·62 to 1·32)	1·41 (0·64 to 3·11)
**Developmental Coordination Disorders, MABC-2 score§**								
mean (SD)	9·6 (3·8)	9·6 (3·8)	8·9 (3·7)	0·49	0·1 (-0·3 to 0·5)	-0·5 (-1·6 to 0·6)	0·1 (-0·3 to 0·6)	-0·2 (-1·3 to 0·9)
≤ 5th percentile ‡	14·0	13·0	23·0	0·24	0·77 (0·44 to 1·34)	2·15 (0·83 to 5·55)	0·77 (0·44 to 1·35)	1·92 (0·73 to 5·03)
**Behavioural difficulties, Total SDQ score**								
mean (SD)	10·6 (5·8)	10·9 (6·2)	12·3 (5·8)	0·027	0·3 (-0·4 to 0·9)	1·4 (-0·0 to 2·8)	0·2 (-0·5 to 0·9)	0·8 (-0·8 to 2·3)
≥ 90th percentile‡	10·3	13·7	12·9	0·18	1·38 (0·95 to 1·99)	1·19 (0·56 to 2·54)	1·36 (0·94 to 1·97)	0·92 (0·41 to 2·08)

Percentages are weighted to consider the differences in survey design between gestational age groups. aOR=adjusted odd ratios; CI=confidence interval; SD=Standard Deviation; GMFCS=Gross Motor Function Classification System; dB=decibel; FSIQ=Full scale intelligence quotient; MABC-2=Movement Assessment Battery for Children- Second Edition; SDQ=Strengths and difficulties questionnaire; GA=Gestational Age.

* Model 1: adjusted for gestational age in week, maternal age at birth, mother birth in France, parents' socio-economic status, cause of preterm birth, antenatal corticosteroids, magnesium sulphate administration, multiple pregnancy, sex, small-for-GA, Apgar at 5 min < 7, birth in type 3 and neonatal unit volume.
Model 2 is model 1 variables and severe neonatal morbidities (late onset sepsis, severe cerebral lesion, severe bronchopulmonary dysplasia, necrotizing enterocolitis, severe retinopathy of prematurity).

† Severe=cerebral palsy gross motor function classification system (GMFCS) level 4/5, and/or bilateral binocular visual acuity < 1/10, and/or uni or bilateral hearing loss > 70 dB, and/or FSIQ < -3 SD; Moderate=cerebral palsy GMFCS level 2/3, and/or 3.2/10 < bilateral binocular visual acuity ≥ 1/10, and/or uni or bilateral hearing loss 40 to 70 dB and/or FSIQ between -3 to-2 SD; Mild=cerebral palsy GMFCS level 1, and/or 5/10 < uni or bilateral binocular visual acuity ≥ 3.2/10, and/or uni or bilateral hearing loss < 40 db, and/or FSIQ between -2 to-1 SD, and/or total MABC-2 score ≤ 5th percentile, and/or behavioural difficulties according to SDQ score ≥ 90th percentile. Cut-off of the distribution related to a reference group born at term.[2,3]

‡ Cut-off of the distribution related to a reference group born at term.[2,3]

§ Among children without cerebral palsy, severe or moderate sensory disabilities, and with full-scale intelligence quotient upper or equal than 2 standard deviations.

Supplementary Figure 1.

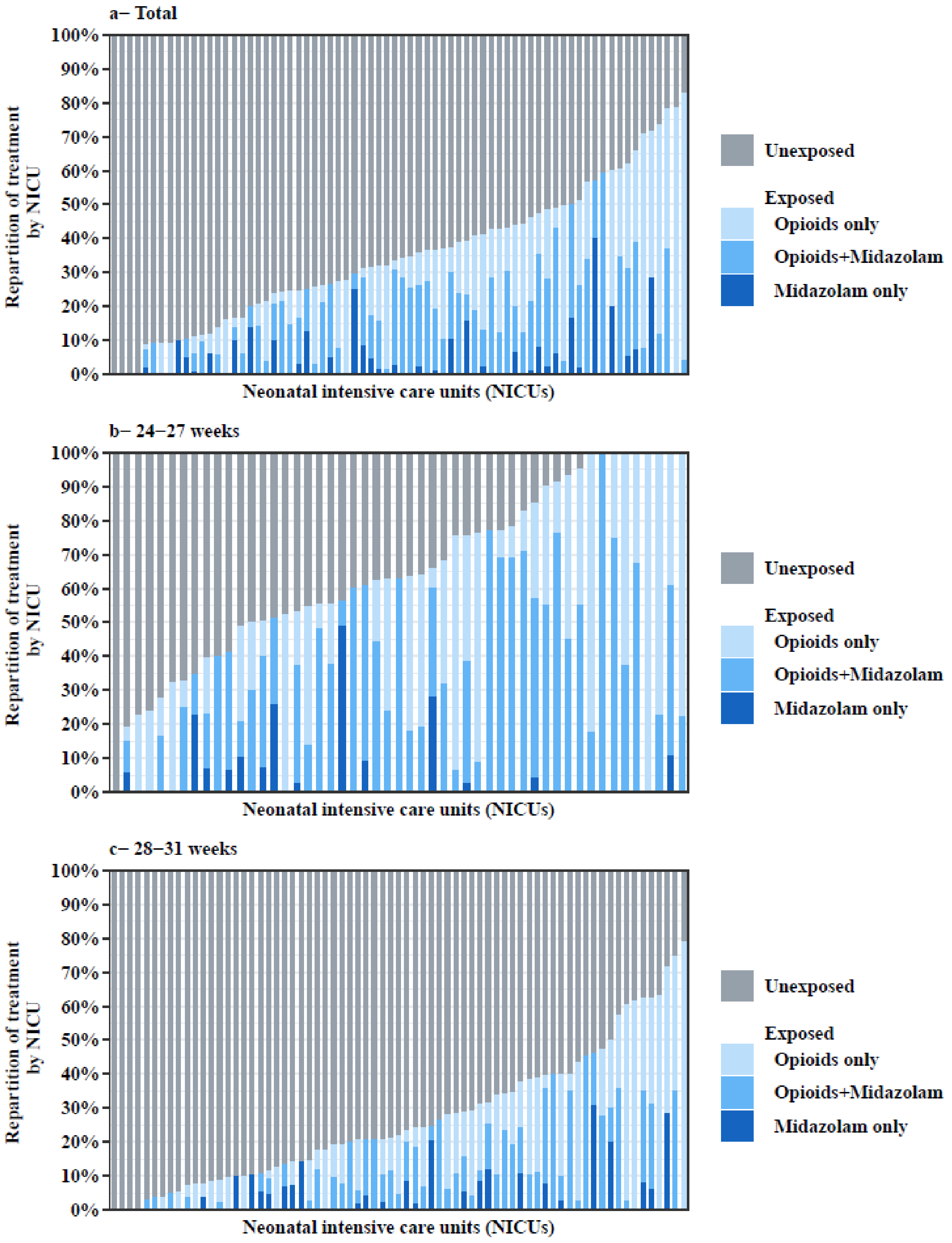

Supplementary references

1. Ego A, Prunet C, Lebreton E, et al. [Customized and non-customized French intrauterine growth curves. I - Methodology]. *J Gynecol Obstet Biol Reprod (Paris)* 2016; **45**(2): 105-64.
2. Pierrat V, Marchand-Martin L, Marret S, et al. Neurodevelopmental outcomes at age 5 among children born preterm: EPIPAGE-2 cohort study. *BMJ* 2021; **373**: n741.
3. Charles MA, Thierry X, Lanoe JL, et al. Cohort Profile: The French national cohort of children (ELFE): birth to 5 years. *Int J Epidemiol* 2020; **49**(2): 368-69j.
