# Supplementary material for: Neurodevelopmental outcomes at five years in children born very preterm (24–31 weeks) exposed to opioids with or without midazolam: results from the French nationwide EPIPAGE-2 cohort study
Source: Lancet Reg Health Eur. 2025 Feb 18;52:101242. doi: 10.1016/j.lanepe.2025.101242 (PMC11883393; doi:10.1016/j.lanepe.2025.101242)
Supplement: Translated abstract [file mmc2.docx]

**Editor Disclaimer:**

This translation in French was submitted by the authors and we reproduce it as supplied. It has not been peer reviewed. Our editorial processes have only been applied to the original abstract in English, which should serve as reference for this manuscript.

**Pré-requis**

Les données épidémiologiques sur le développement neurologique des enfants à l’âge préscolaire, nés prématurément, selon la durée de l’exposition néonatale aux opioïdes avec/sans midazolam, sont limitées.

**Méthodes**

Étude de cohorte prospective incluant des enfants survivants âgés de cinq ans, nés grands prématurés (24-31 semaines) de l'étude nationale française EPIPAGE-2 (Etude Epidémiologique sur les Petits Ages Gestationnels, 2011). L'exposition aux opioïdes avec/sans midazolam a été classée comme absente, ≤ 7 ou >7 jours. Le résultat principal était les troubles neurodéveloppementaux modérés/sévères (TNS). Les pourcentages étaient pondérés pour tenir compte de la conception de l'étude. Les analyses étaient réalisées par des régressions logistiques et ajustées en fonction des facteurs de confusion périnataux.

**Résultats**

Parmi les 3117 survivants, 1165 (35.9%) étaient exposés (762/1165 (68.0%) ≤7 jours, 403/1165 (32.0%) >7 jours). Parmi eux, 49.5% recevaient des opioïdes uniquement, 41.4% des opioïdes et du midazolam, et 9.1% du midazolam uniquement. Un DDN modéré/sévère était observé chez 17.8 %, 18.9 % et 31.7 % des sujets non exposés, exposés ≤ 7 jours et exposés >7 jours, respectivement. Après ajustement sur les facteurs de confusion , seule l'exposition >7 jours était associée à des taux accrus de TNS modéré/sévère (rapport de cotes ajusté 2.07 ; IC à 95 % 1.32-3.26). Après ajustement supplémentaire pour les morbidités néonatales sévères, aucune association significative n'était trouvée entre la durée d'exposition et le DDN, quelle qu'elle soit.

**Interprétation**

L'exposition aux opioïdes avec/sans midazolam > 7 jours pourrait être associée à une prévalence plus élevée de TNS modéré/sévère à cinq ans chez les enfants nés très prématurément, mais les morbidités néonatales sévères sont un modulateur majeur de cette association.

**Financements**

Institut français de recherche en santé publique, Institut national de la santé et de la recherche médicale, Institut national du cancer, Caisse nationale de solidarité pour l'autonomie, fondations PremUp, APICIL.
